# A Novel Urea Complexation Method for Enrichment of n-3 Polyunsaturated Fatty Acids

**DOI:** 10.3390/molecules31091452

**Published:** 2026-04-27

**Authors:** Zhaomin Sun, Feifei Gong, Meng Liu, Ying Li, Guangyu Yan, Lingyu Zhang, Wenqi Zheng, Yanying Tan, Xinyi Peng, Haihua Huang, Hui Ni, Lei Yu

**Affiliations:** 1 School of Marine Biology, Xiamen Ocean Vocational College, Xiamen 361102, China; 2 Applied Technology Engineering Center of Fujian Provincial Higher Education for Marine Food Nutrition Safety and Advanced Processing, Xiamen 361102, China; 3 Xiamen Key Laboratory of Intelligent Fishery, Xiamen 361102, China; 4 Applied Technology Engineering Center of Fujian Provincial Higher Education for Marine Resource Protection and Ecological Governance, Xiamen 361102, China; 5 Fujian Provincial Key Laboratory of Food Microbiology and Enzyme Engineering, Xiamen 361021, China; 6 Research Center of Food Biotechnology of Xiamen City, Xiamen 361021, China; 7 Fujian High Fortune Bio-Tech Corp., Fuzhou 350311, China

**Keywords:** urea complexation, polyunsaturated fatty acids, docosahexaenoic acid, agglomeration, fatty acid ethyl esters, marine oil, processing variable

## Abstract

A novel urea complexation technology was developed based on the agglomeration phenomenon induced by ambient-temperature agitation of a ternary system consisting of urea, water and fatty acid ethyl esters (EEs). The agglomeration phenomenon can be regarded as an intuitive indicator to judge the occurrence of urea complexation. Using docosahexaenoic acid (DHA) containing EE (DHA-EE) from *Crypthecodinium cohnii* oil as the substrate, key variables including agglomeration time, urea/DHA-EE ratio, water/DHA-EE ratio, and temperature were investigated. The urea complexation predominantly occurred within 15 min following agglomerate formation. Temperature in the range of 0–40 °C exerted no significant effect on the yield of the non-urea-complexed fraction or its DHA content, enabling the operation to be conducted at room temperature without heating or cooling. Under unoptimized conditions, the proposed method effectively increased the DHA content of EE from *Crypthecodinium cohnii* oil from 40.73% to 89.87%. For EE from *Schizochytrium* sp. oil, the contents of DHA and docosapentaenoic acid were improved from 47.17% and 13.93% to 69.30% and 20.29%, respectively. Meanwhile, the contents of eicosapentaenoic acid and DHA in two EE form fish oils were enhanced from 18.26% and 11.76% to 34.86% and 22.96%, and from 13.30% and 57.24% to 15.66% and 68.68%, respectively. The present study provided a novel technical pathway for the efficient enrichment of polyunsaturated fatty acids.

## 1. Introduction

N-3 polyunsaturated fatty acids (n-3 PUFA), particularly eicosapentaenoic acid (EPA, C20:5 n-3) and docosahexaenoic acid (DHA, C22:6 n-3), have been well-documented in numerous experimental studies to exhibit a variety of beneficial physiological functions [1,2,3,4,5]. Urea complexation has emerged as a conventional methodology for the enrichment of n-3 PUFA from diverse fatty acid sources [6].

**Figure 1:**
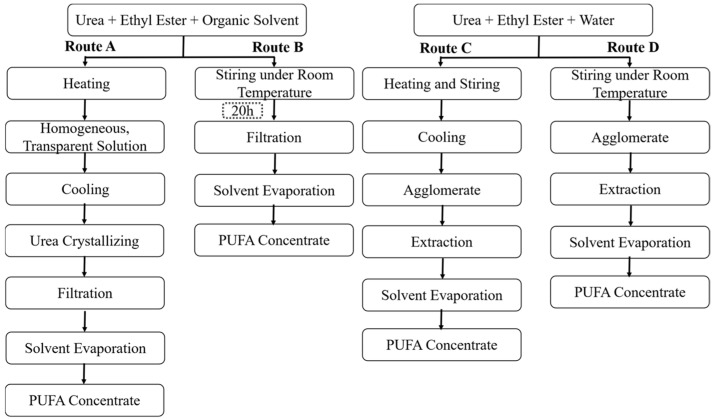
Technical routes of urea complexation.

Classical urea complexation typically includes three sequential steps (Route A in Figure 1). Firstly, urea is dissolved in ethanol with fatty acid ethyl esters (EEs) under elevated temperatures to form a homogeneous, transparent solution [7,8]. Subsequently, the mixture is either subjected to cryogenic cooling [8] or solvent evaporation [9,10] to facilitate the formation of stable urea complexes with saturated and monounsaturation fatty acids. Finally, the resulting slurry is centrifuged or filtered to separate the urea complexes from the solvent phase, and the PUFA-enriched product is then recovered from the polar organic solvent [11]. In this process, high-purity ethanol (>90% *v*/*v*) is generally required to ensure the complete dissolution of EE. However, such high ethanol concentrations conversely reduce the solubility of urea, necessitating higher dissolution temperatures and prolonged processing times. These conditions raise energy consumption and may promote the formation of ethyl carbamate, a potential carcinogen [12].

In recent years, modified urea complexation methods have been developed to improve process efficiency and reduce environmental impact. Notably, previous studies have reported room-temperature urea complexation processes where ethanol was almost completely replaced by hexane (Route B in Figure 1) [12,13]. However, the methods described in these reports still relied on organic solvents throughout the urea complexation process, which typically required up to 20 h to complete.

Our previous study revealed that mixing urea, EE and water at 45–85 °C followed by cooling induced agglomeration, and DHA enrichment occurred concomitantly with this agglomeration process (Route C in Figure 1) [14]. This finding indicated that urea complexation could proceed in the absence of organic solvents, including ethanol, hexane and other food-grade solvents [15]. However, our previous method still required heating and cooling steps, which inevitably led to extra energy consumption.

Inspired by the agglomeration phenomenon of urea upon moisture absorption at room temperature, we subsequently found that gypsum-like solid agglomerates could be obtained by agitating the ternary urea-water-EE system at an ambient temperature. The present study aimed to utilize this ambient-temperature agglomeration phenomenon as an alternative urea complexation strategy for PUFA concentration without any heating or cooling operation (Route D in Figure 1), thus providing a valuable reference for developing a more energy-efficient, low-cost, and safer urea complexation method for PUFA enrichment.

## 2. Results and Discussion

### 2.1. Characterization of the Novel Urea Complexation Process

*Crypthecodinium cohnii* oil has a simple fatty acid profile dominated by DHA, therefore it was selected as the model substrate to investigate the effects of triacylglycerol and EE forms when stirred with urea and water at 30 °C. The lipids were extracted from the resulting mixtures, and their fatty acid composition were subsequently analyzed and presented in Table 1.

**Figure 2:**
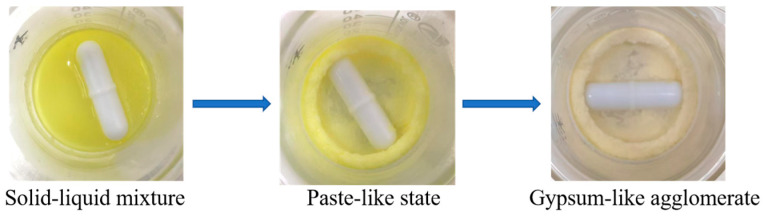
The phase transformation during the novel urea complexation process.

When *Crypthecodinium cohnii* oil EE was stirred with urea and water, the mixture initially formed a solid-liquid heterogeneous system consisting of urea crystals, aqueous urea solution and oil, then transformed into a paste-like state, and finally formed gypsum-like agglomerates (Figure 2). The entire phase change finished within 5 min. Moreover, lipids extracted from the gypsum-like agglomerates showed a significantly higher DHA content than the original EE, confirming that urea complexation occurred during the agglomeration process and achieved PUFA enrichment.

EEs of saturated and monounsaturated fatty acids possess linear carbon chains that can be readily inserted into urea channels to form stable host-guest complexes via hydrogen bonding and van der Waals forces. The formation of intramolecular hydrogen bonds may reduce the solubility of urea complexes, making them insoluble in the low-water-content reaction system and consequently forming agglomerates.

In contrast, the mixture of urea, water and *Crypthecodinium cohnii* oil in triacylglycerol form did not agglomerate even after 4 h of stirring, and the DHA content of the oil remained unchanged post-stirring. This demonstrated that the triacylglycerol form of the algal oil cannot enter the urea channel to form urea complexes. These phenomena suggested that the occurrence of agglomeration during stirring can be used as a visual indicator to judge whether urea complexation took place.

The entire phase transformation process was divided into three stages according to the physical state of the mixture. To determine the time point at which urea complexation initiated, lipids were extracted from the mixture at each stage and their DHA contents were analyzed (shown in Table 2).

As shown in Table 2, the DHA content of lipids extracted from the solid-liquid mixture exhibited no significant change compared with the original EE. However, after the mixture transformed into a paste-like state, the DHA content in the extracted lipids increased from 40.73% to 47.34%, indicating that urea complexation was most likely initiated at this stage.

Once the mixture formed gypsum-like agglomerates, the DHA content of the non-urea-complexed fraction (NUCF) extracted from the agglomerates increased sharply from 50.27% to 85.15% within 15 min, and the concentration factor increased from 1.23 to 2.09 accordingly. With further extension of time, the DHA content continued to increase and reached approximately 95% at 2 h of agglomeration and then kept constant thereafter. These results indicated that the core urea complexation process predominantly occurred within 15 min after agglomerate formation.

One of the key advantages of this novel urea complexation method was the replacement of flammable and explosive organic solvents (e.g., ethanol) with water as the urea-dissolving medium, which could significantly reduce the dosage and consumption of organic solvents throughout the urea complexation process. Furthermore, the method eliminated the need for high-temperature heating to dissolve urea and low-temperature cooling to induce complex formation; gypsum-like solid agglomerates can be formed simply by stirring the ternary system at a mild ambient temperature (30 °C). This not only saved the capital investment in heating and refrigeration equipment but also reduced energy consumption and simplified the industrial production operation process.

### 2.2. Effects of Urea/DHA-EE Ratio, Water/DHA-EE Ratio and Temperature on the Novel Urea Complexation Process

**Figure 3:**
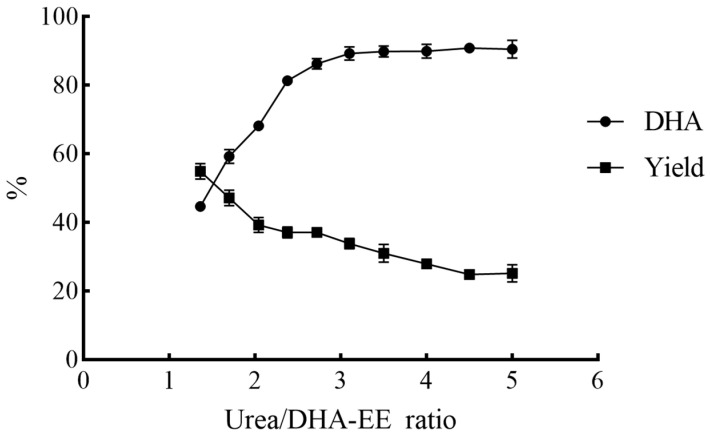
Effects of urea/DHA-EE ratio on the yield and DHA content of NUCF in the novel urea complexation process (experiments were conducted with 1.00 g DHA-EE from *Crypthecodinium cohnii* oil at 30 °C, and the weight ratio of water/DHA-EE was 1.00. NUCF was extracted with petroleum ether 30 min after agglomeration formation).

The urea/EE ratio and cooling crystallization temperature were key parameters governing the traditional urea complexation method [16,17,18,19,20,21,22]. In this study, the effect of Urea/DHA-EE ratio on the novel urea complexation was first investigated at a water/DHA-EE ratio of 1.00 and temperature of 30 °C, and the results were presented in Figure 3.

When the urea/DHA-EE and water/DHA-EE ratios were set at 1.36 and 1.00, respectively, agglomeration occurred after more than 15 min of stirring. When the urea/DHA-EE ratio was increased to 1.70 or higher, gypsum-like agglomerates formed within 15 min of stirring, and the short processing time was favorable for industrial application.

Consistent with the traditional urea complexation method, an increase in the urea/DHA-EE ratio led to an increase in the DHA content of the NUCF but a concomitant decrease in the NUCF yield [16,17,18,19,20,21,22]. The DHA content in NUCF increased from 40.73% to 44.75% at a urea/DHA-EE ratio of 1.36, with a NUCF yield of 53.99%. When the urea/DHA-EE was increased to 2.72, the DHA content in NUCF rose to 81.10%, but the yield decreased to 37.84%. When the urea/DHA-EE ratio reached 3.10, the DHA content in NUCF increased to 89.05%, and further increase in the urea/DHA-EE ratio did not yield additional benefits in terms of DHA content in NUCF.

At a urea/DHA-EE ratio of 1.36, the DHA content of the NUCF increased from the original 40.73% to 44.60%, with a NUCF yield of 54.87%. When the urea/DHA-EE ratio was increased to 2.72, the DHA content of the NUCF rose to 86.18%, while the yield decreased to 37.05%. The DHA content of 89.21% in the NUCF was achieved at a urea/DHA-EE ratio of 3.10, and further increases in the urea/DHA-EE ratio did not result in a significant improvement in the DHA content, indicating that this ratio represented the optimal condition for DHA enrichment.

**Figure 4:**
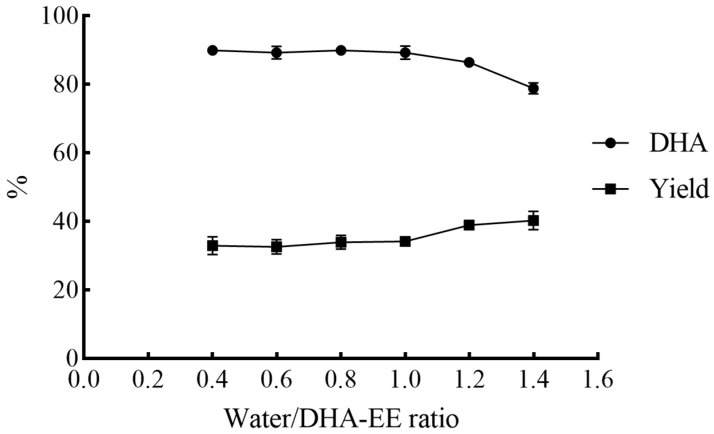
Effects of water/DHA-EE ratio on the yield and DHA content of NUCF in the novel urea complexation process (experiments were conducted with 1.00 g DHA-EE from Crypthecodinium cohnii oil at 30 °C, and the weight ratio of urea/DHA-EE was 3.10. NUCF was extracted with petroleum ether 30 min after agglomeration formation).

Subsequently, the effect of the water/DHA-EE ratio on the novel urea complexation process was investigated at a fixed urea/EE ratio of 3.10 and temperature of 30 °C, and the results were depicted in Figure 4.

When the water/DHA-EE ratio ranged from 0.4 to 1.0, the DHA content of the NUCF remained relatively constant (89.18–89.87%). An increase in the water/EE ratio beyond 1.0 led to a gradual decrease in the DHA content and a simultaneous increase in the NUCF yield, which was likely due to the instability of urea complexes in high-water-content systems, resulting in the partial dissociation of the complexes and the release of non-PUFA fatty acids into the NUCF. Notably, at a water/DHA-EE ratio of 0.4, some undissolved urea crystals were observed that did not participate in agglomerate formation; however, the DHA content of the resulting NUCF was comparable to those obtained at water/EE ratios of 0.6 and 0.8.

Temperature significantly influences urea solubility in water and ethanol [23,24]. In the traditional urea complexation method, it was necessary to heat the urea-ethanol-EE mixture to temperatures above 60 °C to obtain a homogeneous solution [18,22], which was then cooled to below room temperature to induce urea crystallization and complex formation. Generally, the lower the cooling temperature, the more urea precipitated out of the solution and participated in the complexation process, which was conducive to increasing the PUFA content in the NUCF.

In our previous study [14], agitation of a ternary system consisting of urea, water and EE with heating followed by cooling also induced agglomeration, and the DHA content in NUCF was significantly affected by the heating temperature (45–85 °C) of the mixture, but not by the cooling temperature (0–40 °C).

**Figure 5:**
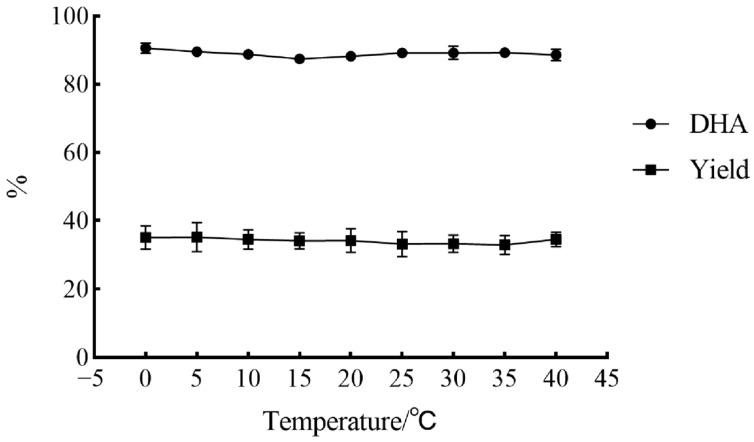
Effects of temperature on the yield and DHA content of NUCF in the novel urea complexation process (experiments were conducted with 1.00 g DHA-EE from *Crypthecodinium cohnii* oil, and the urea/DHA-EE ratio and water/DHA-EE ratio were 3.10 and 1.00, respectively. The NUCF was extracted with petroleum ether 30 min after agglomeration formation).

In the present study, the effect of temperature on the novel urea complexation process was examined at a urea/DHA-EE ratio of 3.10 and water/DHA-EE ratio of 0.80, and the results were shown in Figure 5. Temperature in the range of 0–40 °C exerted no significant effect on either the DHA content or the yield of the NUCF, indicating that temperature was not a critical parameter for this novel urea complexation method. The entire agglomeration process can be realized by stirring at ambient temperature (0–40 °C) without any heating or cooling treatment, which fundamentally simplified the process, reduced energy consumption and improved operational safety compared with our previous study [14]. The novel method conferred significant advantages in terms of energy conservation and likely in reduction in ethyl carbamate formation compared with traditional methods [12,15].

### 2.3. Applicability of the Novel Urea Complexation Method to Different PUFA-Containing EE

To evaluate the general applicability of the novel urea complexation method, the process was applied to different PUFA-containing EEs (from *Schizochytrium* sp. oil and two fish oils), and the enrichment results are presented in Table 3.

As shown in Table 3, the contents of the target PUFA (DHA, EPA, DPA) in all tested EE sources were significantly increased after treatment with the novel urea complexation method, which confirmed the broad applicability of this technology to different PUFA-containing raw materials. Notably, the data shown in Table 3 and the enrichment results for DHA-EE from *Crypthecodinium cohnii* oil discussed above were acquired under non-optimized conditions. Further systematic optimization by response surface methodology is expected to achieve an even higher PUFA content in the NUCF.

As depicted in Table 3, the concentration factors differed among various kinds of EEs and among identical EEs from different sources after urea complexation treatment. Feedstocks with higher PUFA content had lower concentration factors because fewer saturated fatty acids were available to form stable complexes and obvious agglomerates.

### 2.4. Limitations and Application Prospects of the Novel Urea Complexation Method

As previously demonstrated, the occurrence of agglomeration during stirring can be used as a visual indicator to judge the progress of urea complexation. When EEs from *Schizochytrium* sp. oil, *Crypthecodinium cohnii* oil and fish oil A were treated at a urea/EE ratio of 3.10 and a water/EE ratio of 0.80, distinct agglomeration was observed within 15 min of stirring. The agglomeration observation is simple to operate for real-time monitoring and does not require complex instruments, which provides convenience for quality control in industrial production.

However, the difficulty of forming gypsum-like agglomerates with urea and water increased significantly when the raw material EE contained a high proportion of PUFA. The bent structure of PUFA prevented them from entering urea’s hexagonal channel structure, and hindered the formation of stable inclusion complexes. Higher PUFA content in the substrate would result in fewer inclusion complexes being formed and subsequent weak or indistinct agglomeration behavior. Taking fish oil B (13.30% EPA, 57.24% DHA) as an example, no significant agglomeration was observed even after 6 h of stirring at a urea/EE ratio of 3.10, a water/EE ratio of 1.00 and a temperature of 30 °C. When the reaction conditions were adjusted to a higher urea/EE ratio (4.00) and a lower water/EE ratio (0.40), faint agglomeration became observable after 4 h of continuous stirring. These results indicated that for PUFA-enriched feedstock oils, increasing the urea/EE ratio and decreasing the water/EE ratio could accelerate the urea complexation process and promote agglomeration. However, when NUCF with a high DHA content (87.14%) was subjected to a second round of complexation at a urea/EE ratio of 5.00 and a water/EE ratio of 0.60, no effective agglomeration was formed even after 24 h of continuous stirring.

The results above indicated that the novel urea complexation method shared the same limitations encountered in conventional urea complexation approaches. They cannot achieve further enrichment of PUFA once the PUFA content in the oil reached a certain threshold, because highly enriched PUFA with bent, irregular conformation alkyl chains had a low tendency to form urea complexes.

Although the novel urea complexation method used no organic solvents, petroleum ether was still needed to extract NUCF from the agglomerate. This may cause environmental, production safety and food safety concerns. In scaled up operations, the novel method may face issues such as uniformity of stirring, low NUCF extraction efficiency from agglomerates, and unstable process performance.

Despite the above limitations, the novel urea complexation method still has broad application prospects.

The avoidance of organic solvents during the core urea complexation process still has obvious advantages in reducing environmental pollution and production costs compared with traditional methods that use organic solvents throughout the process. The residual solvent in NUCF can be effectively removed through thin-layer vacuum evaporation to meet the relevant food safety standards.

The novel method is characterized by simple operational procedures and reduced energy consumption. Moreover, it demonstrates excellent industrial applicability and popularization potential, which is conducive to the large-scale industrial application and promotion of this technology.

## 3. Material and Methods

### 3.1. Material

Natural DHA-rich microalgal oils from *Crypthecodinium cohnii* and *Schizochytrium* sp., both in triacylglycerol form, were purchased from Henan Hengkang Biotech Co., Ltd. (Zhengzhou, China) and Qingdao Xu’neng Bioengineering Co., Ltd. (Qingdao, China), respectively. Commercial fish oil A (18.26% EPA, 11.76% DHA) and fish oil B (13.30% EPA, 57.24% DHA), both in EE form, were supplied by Fujian High Fortune Bio-Tech Co., Ltd. (Fuzhou, China). All solvents and chemical reagents used were of analytical grade and obtained from Xilong Scientific Co., Ltd. (Shantou, China).

### 3.2. The Preparation of DHA-EE

DHA-EE was prepared via alcoholysis of *Crypthecodinium cohnii* oil as follows: 250 g of *Crypthecodinium cohnii* oil was mixed with 100 mL of anhydrous ethanol and 2.5 g of NaOH. The mixture was magnetically stirred for 2 h at 55 °C under a nitrogen atmosphere, then transferred to a separatory funnel and allowed to stratify at room temperature overnight. The upper EE phase was washed three times with 5% (*w*/*w*) NaCl solution at 80 °C and dehydrated with anhydrous sodium sulfate to obtain DHA-EE with a purity of 98% (*w*/*w*).

The DHA-EE obtained from *Crypthecodinium cohnii* oil contained 40.73% DHA, while the DHA-EE derived from *Schizochytrium* sp. oil contained 13.93% docosapentaenoic acid (DPA, C22:5 n-6) and 47.17% DHA.

### 3.3. Urea Complexation

Urea, water and algal oil EEs were accurately weighed and transferred into a screw-cap glass vial. The vial was placed in a thermostatic magnetic stirrer, and the mixture was stirred at 250 rpm, during which solidification and agglomeration of the mixture were observed. The solid agglomerate was collected, crushed manually, and then extracted with petroleum ether. The extract was filtered to remove residual solid impurities, and the filtrate was subjected to reduced-pressure rotary evaporation to remove petroleum ether, yielding the NUCF.

The yield of NUCF was calculated according to the following equation:

$$Yield(\%)=\frac{W_{NUCF}}{W_{EE}}\times \;100\%$$

where *W_NUCF_* was the weight of the obtained NUCF and *W_EE_* was the weight of the DHA-EE raw material used in the urea complexation experiment.

The concentration factor was calculated according to the following formula:

$$Concentration\;factor=\frac{Content\;of\;a\;specific\;fatty\;acid\;in\;concentrate}{Content\;of\;a\;specific\;fatty\;acid\;in\;substrate}$$

### 3.4. The Methylation of Fatty Acids

Approximately 10 mg of the oil sample (DHA microalgal oil, DHA-EE, fish oil or NUCF) was placed in a screw-cap glass tube, and 2 mL of 10% (*v*/*v*) sulfuric acid-methanol solution was added. The tube was purged with nitrogen, sealed, and heated at 60 °C for 15 min to facilitate fatty acid methylation. After cooling to room temperature, petroleum ether was added to extract the formed fatty acid methyl esters (FAMEs), which were subsequently used for fatty acid composition analysis by gas chromatography (GC).

### 3.5. The Fatty Acid Composition Analysis

FAME analysis was performed using an Agilent 7890A gas chromatograph (Agilent Technologies Inc., Santa Clara, CA, USA) equipped with a hydrogen flame ionization detector (FID) and a BP-Inowax capillary column (30 m × 0.32 mm × 0.25 μm, Nano Spectrum Analytical Technology Co., Ltd., Suzhou, China). The column temperature was ramped from 170 °C to 210 °C at 3 °C/min, and held at 210 °C for 30 min. Nitrogen was used as the carrier gas at a constant flow rate of 1 mL/min with a split ratio of 25:1. The injector and FID temperatures were set at 250 °C and 280 °C, respectively. The fatty acid composition of each sample was quantified by the peak area normalization method according to the following equation:

$$Y_{i}(\%)=\frac{A_{i}\times F_{i}}{\sum A_{i}\times F_{i}}\times \;100\%$$

where *Y_i_* was the weight percentage of a specific fatty acid relative to the total fatty acids in the sample, *A_i_* presented the peak area of each FAME determined in the sample analysis; and *F_i_* was the conversion coefficient for transforming a FAME into its corresponding free fatty acid in the sample.

### 3.6. Statistical Analysis

All experiments were performed in triplicate. Statistical analysis was conducted using GraphPad Prism^®^ Version 8.02 (GraphPad Software, Inc., San Diego, CA, USA). Experimental data are expressed as the mean ± standard deviation (SD). Differences among group means were compared using one-way analysis of variance (ANOVA) followed by the Tukey–Kramer multiple comparison test. A *p*-value < 0.05 was considered statistically significant.

## 4. Conclusions

In this study, a novel urea complexation method was developed for efficient enrichment of n-3 PUFA. It relied on agglomeration by stirring a ternary system of urea, water and EE at room temperature. The agglomeration of the reaction mixture can be used as a simple, intuitive visual indicator to judge the occurrence of urea complexation. The core urea complexation reaction was concentrated within 15 min following agglomerate formation.

The parameters affecting the efficiency of this novel method were the urea/EE ratio and water/EE ratio. Temperature in the range of 0–40 °C exerted no significant effect on the complexation efficiency, enabling the method to be operated at room temperature without the need for heating or cooling equipment. Feasibility verification experiments confirmed that this method was applicable to a variety of PUFA-containing EE raw materials and can effectively increase the contents of functional PUFA.

In comparison with traditional urea complexation technologies, the novel method eliminated the requirement for large volumes of flammable and explosive organic solvents, simplified the heating-cooling process, and offered the distinct advantages of energy conservation, low production cost, high operational safety and environmental friendliness. Thus, this novel urea complexation method is a practical and promising option for industrial enrichment of n-3 PUFA, with wide applications in food, supplements and pharmaceuticals.

## Figures and Tables

**Table 1:** Results after stirring different chemical forms of *Crypthecodinium cohnii* oil with urea and water.

*Crypthecodinium cohnii* Oil Form	Phenomena After Stirring	DHA Content of the Extracted Lipids (%)
Triacylglycerols	A yellow heterogeneous mixture of urea crystals, saturated urea solution and oil was formed; no agglomeration was observed even after 4 h of stirring.	40.26 ± 0.24
EEs	Pale yellow gypsum-like agglomerates formed within 5 min of stirring.	50.27 ± 0.96

Experiments were conducted with 1.00 g of *Crypthecodinium cohnii* oil at 30 °C; the weight ratios of urea to oil and water to oil were 2.72 and 1.00, respectively. Petroleum ether extraction was performed immediately after agglomeration formation (for EEs) or after 4 h of stirring (for triacylglycerols).

**Table 2:** DHA content of lipids extracted from the mixture of urea, water and DHA-containing EE (DHA-EE) at different physical states and agglomeration durations.

Mixture State	Agglomeration Duration (h)	DHA Content (%)	Concentration Factor of DHA
Solid–liquid mixture	-	40.67 ± 0.12	-
Paste-like state	-	47.34 ± 1.61	1.16
Gypsum-like agglomerate	0	50.27 ± 0.96	1.23
	0.25	85.15 ± 1.49	2.09
	0.50	86.18 ± 1.23	2.12
	1.0	92.63 ± 1.44	2.27
	1.5	94.39 ± 2.07	2.32
	2.0	95.44 ± 1.03	2.34
	3.0	95.72 ± 1.88	2.35
	4.0	95.63 ± 2.12	2.35

Experiments were conducted with 1.00 g of DHA-EE from *Crypthecodinium cohnii* oil at 30 °C, and the weight ratios of urea/DHA-EE and water/DHA-EE were 2.72 and 1.00, respectively. Petroleum ether extraction was performed immediately after the formation of the paste-like state or gypsum-like agglomerates.

**Table 3:** Enrichment of target PUFA from different EE sources using the novel urea complexation method.

	Materials (%)	NUCF (%)	Concentration Factor
EE from *Schizochytrium* sp. oil *	DPA 13.93 ± 0.01, DHA 47.17 ± 0.21	DPA 20.29 ± 0.34, DHA 69.30 ± 0.23	1.49 1.47
Fish oil A*	EPA 18.26 ± 0.25, DHA 11.76 ± 0.16	EPA 34.86 ± 1.36, DHA 22.96 ± 1.30	1.81 2.11
Fish oil B **	EPA 13.30 ± 0.33, DHA 57.24 ± 0.29	EPA 15.66 ± 0.35, DHA 68.68 ± 0.88	1.19 1.19

* Experiments were conducted with 1.00 g of EE at room temperature, and the urea/EE ratio and water/EE ratio were 3.10 and 0.80, respectively. The NUCF was extracted with petroleum ether 30 min after agglomeration formation. ** Experiments were conducted with 1.00 g EE at room temperature, and the urea/EE ratio and water/EE ratio were 4.00 and 0.40, respectively. The NUCF was extracted with petroleum ether 30 min after agglomeration formation.

## Data Availability

The datasets generated for this study are available on request to the corresponding author.

## Abbreviations

The following abbreviations are used in this manuscript:

DHA	Docosahexaenoic acid
DPA	Docosapentaenoic acid
EE	Fatty acid ethyl esters
EPA	Eicosapentaenoic acid
FID	Flame ionization detector
GC	Gas chromatography
NUCF	Non-urea-complexed fraction
PUFA	Polyunsaturated fatty acids
SD	Standard deviation
